# Misleading Presentation: Chest Pain Masking Euglycemic Diabetic Ketoacidosis Possibly Induced by Empagliflozin

**DOI:** 10.7759/cureus.49402

**Published:** 2023-11-25

**Authors:** Kripa Tiwari, Nava R Sharma, Madalasa Pokhrel, Arjun Basnet, Michael Kaplan

**Affiliations:** 1 Internal Medicine, Maimonides Medical Center, Brooklyn, USA; 2 Medicine, Manipal College of Medical Science, Pokhara, NPL; 3 Internal Medicine, Montefiore Medical Center, New Rochelle, USA

**Keywords:** non-st segment elevation myocardial infarction (nstemi), atypical chest pain, sglt-2 inhibitor, precipitating factors for euglycemic dka, diabetic ketoacidosis (dka), dka

## Abstract

Diabetic ketoacidosis (DKA) is a life-threatening metabolic emergency traditionally associated with Type 1 diabetes but is increasingly recognized in Type 2 diabetes, particularly with the use of sodium-glucose cotransporter-2 (SGLT-2) inhibitors. Euglycemic DKA, characterized by near-normal blood glucose levels, is a distinct variant that has gained attention. This case report highlights a unique presentation of euglycemic DKA in a 56-year-old female with a past medical history of Type 2 Diabetes Mellitus who presented to the emergency department with a one-week history of chest pain.

## Introduction

Diabetic ketoacidosis (DKA) is a severe metabolic emergency characterized by hyperglycemia, ketosis, and metabolic acidosis, which has traditionally been associated with individuals diagnosed with Type 1 diabetes [1]. However, there is a growing recognition of its occurrence in individuals with Type 2 diabetes, primarily because of the increased use of sodium-glucose cotransporter-2 (SGLT-2) inhibitors [1]. These medications, mainly prescribed for the management of Type 2 diabetes, have led to a notable increase in the incidence of DKA, with a particular focus on euglycemic DKA, a distinct presentation marked by blood glucose levels within the normal range. Empagliflozin, a well-known SGLT-2 inhibitor, has demonstrated significant advantages in glycemic control and cardiovascular outcomes for individuals with Type 2 diabetes [2].

However, there is a growing concern regarding the potential of SGLT-2 inhibitors to induce DKA, including the euglycemic variant. Despite ongoing research, the precise mechanisms responsible for this phenomenon remain incompletely understood. This case report underscores the critical importance of staying vigilant when evaluating patients with Type 2 diabetes, especially those utilizing SGLT-2 inhibitors, who present with unusual symptoms such as chest pain.

## Case presentation

A 56-year-old female with a past medical history of type 2 diabetes mellitus (T2DM) presented to the emergency department (ED) with a one-week history of chest pain and generalized weakness. The chest pain presented as an acute-onset, retrosternal discomfort with a constant nature, non-radiating pattern, and heightened intensity during physical activity, particularly exacerbated by walking distances as short as half a block. The patient denied experiencing fever, shortness of breath, orthopnea, palpitation, leg swelling, leg claudication, nausea, vomiting, or abdominal pain.

During further inquiry regarding her medication history, the patient disclosed that she had been newly diagnosed with diabetes mellitus about one year back, which was accompanied by an episode of DKA and pancreatitis that was suspected to be associated with COVID-19 infection. Following her initial diagnosis, she was discharged on insulin therapy, which she continued for three months. Her endocrinologist later transitioned her to oral medications due to improvements in her pancreatic function, prescribing glimepiride 4mg twice daily, metformin 1gm twice daily, and Empagliflozin 25 mg (taking half a tablet daily).

Upon initial assessment, the patient exhibited a blood pressure reading of 107/68 mmHg, a heart rate of 85 beats per minute, a respiratory rate of 15, saturation levels exceeding 95%, and maintained afebrile status. Systemic examination revealed unremarkable findings, including bilateral air entry without added sounds, normal S1 and S2 heart sounds, and the absence of murmurs.

**Figure 1:**
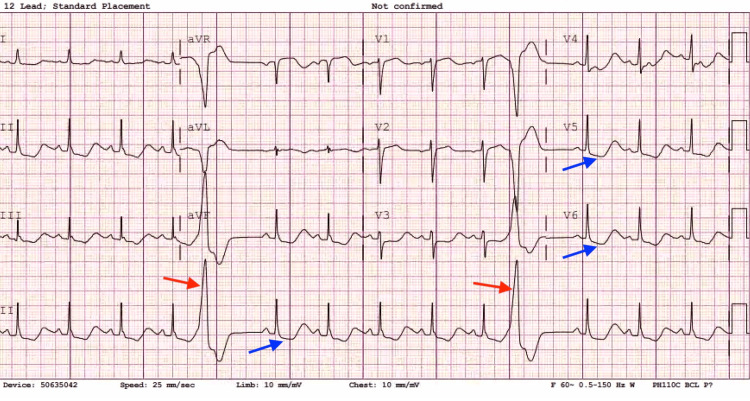
Electrocardiogram displaying frequent Premature Ventricular Contractions (PVCs) as shown by red arrows and ST-segment depression shown by blue arrows.

The initial investigations in the ED revealed an electrocardiogram (EKG) displaying normal sinus rhythm at a rate of 85 beats per minute, frequent premature ventricular contractions (PVCs), and ST-segment depression in leads V4-V6 as well as anterolateral leads, as shown in Figure 1. Troponin levels were found to be 0.01 ng/mL (normal reference range: 0.00-0.04 ng/ml). The echocardiogram revealed normal left ventricular systolic function with no wall motion abnormality and an ejection fraction (EF) of 56-60%.

Considering the concerning EKG findings, cardiology was consulted, and the patient was immediately transported to the catheterization laboratory. The left heart catheterization performed in the Cath lab revealed non-obstructive coronary artery disease (CAD) with a 30% stenosis in the left anterior descending (LAD) artery. The patient was managed under the presumption of myocardial infarction with non-obstructive coronary arteries (MINOCA) with aspirin and statin as per cardiology recommendation.

Concurrently, laboratory results, including a complete metabolic profile, showed glucose levels of 173 mg/dL, sodium at 135 mEq/L, potassium at 2.5 mEq/L, bicarbonate at 13 mEq/L, and an anion gap of 31 mEq/L. Additionally, the patient's glycosylated hemoglobin (HbA1c) level was 7.1 %. A venous blood gas analysis (VBG) showed a pH of 7.28, pCO_2_ of 32.2 mmHg, lactic acid of 3.1, and bicarbonate levels at 15.1 mmol/L. Notably, blood culture results showed no growth of organisms, and the white blood cell count was normal. Additionally, the N-terminal prohormone of brain natriuretic peptide (NT-pro BNP) or brain natriuretic peptide (BNP) levels were within the normal range. The chest X-ray revealed no abnormalities. Laboratory values are tabulated in Table 1 below.

**Table 1:** Laboratory values.

Laboratory Value	Result	Normal Range
Glucose	173 mg/dL	70 - 100 mg/dL
Sodium	135 mEq/L	135 - 145 mEq/L
Potassium	2.5 mEq/L	3.5 - 5.0 mEq/L
Bicarbonate (HCO_3_)	13 mEq/L	22 - 28 mEq/L
Anion Gap	31 mEq/L	8 - 16 mEq/L
Glycosylated Hemoglobin	7.10%	4.0 - 5.6%
pH (Venous Blood Gas)	7.28	7.35 - 7.45
pCO_2_ (Venous Blood Gas)	32.2 mmHg	35 - 45 mmHg
Beta-Hydroxybutyrate	0.30 mmol/L	0.02 - 0.27 mmol/L
GAD Antibody	0.05	<5.0 units (Negative)
Pancreatic Antibody	<1:4	<1:4 (Negative)

**Figure 2:**
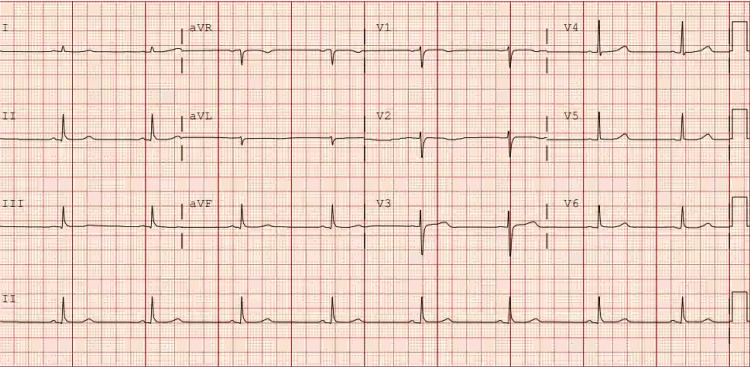
Repeat EKG showing resolution of ST-segment depression and PVCs.

The patient was admitted to the critical care unit with a diagnosis of probable euglycemic DKA. Aggressive resuscitation measures were implemented, involving intravenous fluid administration, potassium repletion, and the initiation of an insulin drip. After treating DKA and correcting electrolyte abnormalities, the chest pain resolved. We also conducted another EKG on the third day of admission, shown in Figure 2, which showed the complete resolution of previous EKG changes.

Throughout her hospital stay, a complete metabolic panel and venous blood gas parameters were monitored every three hours, and electrolyte imbalances were corrected accordingly. On the third day of admission, the anion gap decreased to 9 with a measured beta-hydroxybutyrate level of 0.30 mmol/L. The VBG analysis displayed a pH of 7.44, bicarbonate levels of 30.5 mmol/L, and pCO_2_ at 48.3 mmHg. The patient was transitioned to subcutaneous insulin therapy, with insulin Lispro administered at 2 units every four hours. She tolerated her diabetic diet well, and Glargine at a dose of 15 units every morning (qAM) was added to her treatment regimen. Consultation with the endocrinology team was done, and the patient was subsequently downgraded from the critical care unit to the general medicine floor. Further workup, including testing for islet cell antibodies and glutamic acid decarboxylase (GAD) antibodies, was conducted. Laboratory results revealed a GAD antibody level of 0.05 and a pancreatic antibody level of less than 1:4. 

Dietary recommendations included abstaining from a ketogenic diet and adopting a time-restricted eating pattern. Upon discharge, the patient was instructed to continue her insulin regimen, consisting of Glargine at 15 units qAM and Lispro of 4 units three times daily before meals. She was advised to continue with aspirin and a statin per cardiology recommendations and to follow up with the cardiologist and endocrinologist in the outpatient setting.

## Discussion

DKA is a hyperglycemic emergency characterized by the triad of hyperglycemia, ketosis, and metabolic acidosis with an elevated anion gap. It can serve as the initial manifestation in approximately 25-40% of individuals with type 1 diabetes and is also noted in those with type 2 diabetes [3,4]. The introduction of SGLT-2 inhibitors, primarily prescribed for managing T2D, has led to an increase in the incidence and prevalence of DKA, particularly euglycemic DKA. However, it's important to note that DKA associated with SGLT2 inhibitor use is much less common in T2D, with an incidence of less than 0.2% [5].

Euglycemic DKA is a distinct variant of DKA characterized by the absence of markedly elevated blood glucose levels, typically presenting with a range of 200-300 mg/dL or even lower [6]. It is most associated with the use of sodium-glucose cotransporter-2 (SGLT-2) inhibitors. Empagliflozin, an SGLT-2 inhibitor, has significantly improved glycemic control and cardiovascular outcomes for patients with T2D. However, an emerging concern is its potential to induce DKA, including euglycemic DKA. Several cases in the literature have linked empagliflozin use to this condition. The mechanism behind the unusual presentation of diabetic ketoacidosis in patients taking SGLT-2 inhibitors remains incompletely understood, but several explanations have been proposed [7,8]. SGLT-2 inhibitors effectively lower blood glucose levels by reducing insulin secretion from pancreatic β-cells and potentially increasing glucagon release from α cells. This combination of elevated glucagon and reduced insulin levels contributes to heightened lipolysis and ketogenesis [7,8]. It's essential to note that while the reduction in insulin and increase in glucagon may contribute to heightened lipolysis and ketogenesis, the primary glucose-lowering effect of SGLT-2 inhibitors is through increased urinary glucose excretion [7,9]. This unique mechanism differentiates them from other antidiabetic medications. The exact incidence and prevalence of empagliflozin-induced DKA remain to be fully elucidated [7].

Our patient's transition from insulin therapy to oral antidiabetic agents, including empagliflozin, might have been a contributing factor in the development of euglycemic DKA. Additionally, her prior episode of pancreatitis, possibly linked to a COVID-19 infection, could have compromised her pancreatic function, leading to DKA susceptibility [10]. The clinical presentation of DKA typically includes symptoms like polyuria, polydipsia, vomiting, abdominal pain, and altered mental status [4,5]. The underlying causes of these disturbances remain ambiguous. Factors such as gastric dilatation, paralytic ileus, pancreatitis, or tension on the liver capsule or liver infarct might contribute to these issues [4,11]. The occurrence of chest pain possibly induced by lactic acidosis is an unusual presentation [11].

Treatment of euglycemic DKA and hyperglycemic DKA primarily involves fluid resuscitation, correction of electrolyte imbalances, and insulin administration. The main difference lies in recognizing the near-normal glucose levels in euglycemic DKA and the need for specific laboratory tests, such as beta-hydroxybutyrate measurements, to confirm the diagnosis. It is critical to differentiate between the two forms of DKA to initiate the appropriate treatment and ensure patient safety.

This case report underscores the importance of vigilance and a high index of suspicion when evaluating patients with T2D, particularly those using SGLT-2 inhibitors, who present with cardiac symptoms such as chest pain. Early diagnosis and appropriate management are crucial in mitigating the morbidity and mortality associated with euglycemic DKA. Further research is needed to better understand the incidence, prevalence, and risk factors associated with euglycemic DKA, especially in the context of SGLT-2 inhibitor use.

## Conclusions

This case emphasizes the importance of considering euglycemic diabetic ketoacidosis in patients with T2DM, particularly those on sodium-glucose cotransporter-2 (SGLT-2) inhibitors, when they present with atypical symptoms like chest pain. Euglycemic DKA, though rare, demands a high index of suspicion due to its potentially life-threatening nature. Early recognition, diagnosis, and appropriate management are critical for better patient outcomes. Clinicians should be aware of the unique features of euglycemic DKA and its distinction from hyperglycemic DKA, with normal or near-normal glucose levels. Prompt intervention, including fluid resuscitation, electrolyte correction, and insulin therapy, is essential. Patient history, medication changes, and risk factors, such as infections or fasting, should be thoroughly considered when evaluating these cases. This report underscores the need for vigilance in diagnosing and managing euglycemic DKA, especially among patients with T2DM who use SGLT-2 inhibitors. Further research is warranted to better understand its incidence, prevalence, and associated risk factors.
